# Individual use of self-medication and other remedies in COVID-19 outpatients in Western-Pomerania

**DOI:** 10.1038/s41598-024-72440-w

**Published:** 2024-09-20

**Authors:** Svea Stüdemann, Eik Schäfer, Klaus Hahnenkamp, Mladen V. Tzvetkov, Stefan Engeli

**Affiliations:** 1 Clinical Pharmacology, Institute of Pharmacology, Center of Drug Absorption and Transport (C_DAT), University Medicine Greifswald, Greifswald, Germany; 2 Department of Anesthesia, Intensive Care, Emergency and Pain Medicine, University Medicine Greifswald, Greifswald, Germany; 3 General Pharmacology, Institute of Pharmacology, Center of Drug Absorption and Transport (C_DAT), University Medicine Greifswald, Greifswald, Germany

**Keywords:** COVID-19, Outpatients, Self-medication, OTC, Herbal remedies, Phytopharmaceuticals, Drug therapy, Health care, Medical research

## Abstract

We analyzed data from positively tested COVID-19 outpatients to describe self-medication with OTC drugs and use of other remedies against symptoms of SARS-CoV-2 infection. We specifically considered their type and frequency, as well as associations with patient characteristics, and reasons for use. Data were collected between May 1, 2020 and February 22, 2021 with two questionnaires in an observational cohort study with PCR-confirmed SARS-CoV-2-positive adult outpatients in the district of Western Pomerania in Germany. 523 out of 710 outpatients (74%; 340 women and 183 men) reported using drugs and other remedies to relieve COVID-19-symptoms. Overall, participants reported utilization of 1282 finished dosage products or remedies, including 213 different ingredients. In the population of 710 outpatients, utilization of ibuprofen (26%), acetaminophen (21%), metamizole (14%), and acetylsalicylic acid (10%) was most commonly reported. Phytopharmaceuticals, herbal and animal products as well as vitamins and minerals were also frequently reported. Among the 523 participants who used drugs and other remedies, most commonly mentioned reasons for use were headache (40%), other kinds of pain (e.g. myalgia; 37%), fever (24%) and cough (16%). Our analysis showed that a majority of the participants tried to alleviate COVID-19-symptoms using drugs and other remedies. Especially analgesic and antipyretic agents, followed by herbal medicines, were used very frequently.

*Trial registration:* German Register for Clinical Studies DRKS00021672, first registration on December 1st, 2020.

## Introduction

In January 2020, the first case of SARS-CoV-2 infection was registered in Germany. Since March 2020, the World Health Organization has declared this outbreak as a pandemic^1^. Since COVID-19 is a new disease, knowledge about the course, therapy, and general management had to and still has to develop. About 10% of infected patients require hospitalization^2^. Consequently, the majority of COVID-19 patients seeking help are outpatients. Since December 2020, vaccinations against COVID-19 are available in Germany and protect against severe disease and death^3^. Nevertheless, vaccination does not protect from infection and transmission, therefore the need for effective medicines persists. So far, only tocilizumab, remdesivir and the combination of nirmatrelvir + ritonavir received a marketing authorisation for use in the European Union to treat COVID-19 and are also recommended in the German living guideline for treatment of patients with COVID-19^4,5^. Molnupiravir was allowed for treatment by the Federal Ministry of Health in Germany without regulatory approval. In February 2023, the Committee for Medicinal Products for Human Use of the European Medicines Agency recommended refusing the marketing authorisation, and based on this assessment the company withdrew its application^6^. Until the end of the recruitment period, the mentioned specific therapeutic options for outpatients, such as monoclonal antibodies, nirmatrelvir + ritonavir, or molnupiravir were not yet available. The aim of this analysis was to determine the use of drugs and other remedies against symptoms of SARS-CoV-2 infection in outpatients. In particular, we were interested to record the active ingredients and the number of participants using these, as well as examine associations with patient characteristics and reasons for utilization. To the best of our knowledge, there are no other studies with a similarly detailed and comprehensive description of the use of drugs and other remedies, especially on self-medication, in outpatients with SARS-CoV-2 infection confirmed by positiv PCR-test. In particular several studies analyzed self-medication during COVID-19 pandemic, regardless of whether a disease was present or not, and recruited mostly in countries other than Germany or from Europe^7–10^. Moreover studies conducted in countries other than Germany often show a high number of drugs used in self-medication that are only available on prescription in Germany. Our analysis could help to give an overview of the drugs, especially over-the-counter (OTC) drugs, and other remedies used and to provide a basis for further studies examining needs and challenges in treatment and counseling of COVID-19 outpatients.

## Materials and methods

This analysis based on the data collection for the COVER study by our group^11^. Between May 1, 2020 and February 22, 2021, in collaboration with the health department of the district of Vorpommern-Greifswald (Western Pomerania, Germany), we invited adult outpatients with a first positive PCR (polymerase chain reaction)-confirmed SARS-CoV-2 infection to participate in this observational cohort study. We did not invite individuals younger than 18 years or with a positive PCR-test result older than one week. The conduct of the study was in accordance with the Declaration of Helsinki 2013 and approved by the Ethics Committee of the University Medicine Greifswald (internal ethics committee registration BB 059/20). The study is registered in the public German Register for Clinical Studies (DRKS00021672).

Each positiv PCR-test result in Vorpommern-Greifswald (e.g. from public test centers or medical practices) had to be reported to the local health department. Due to our collaboration we were able to contact all the individuals with a positive test result. The invitations were sent by mail and included paper questionnaires, information sheeds about the study, the informed consent form as well as prepaid and pre-addressed envelopes to return the documents. Study participants were asked to complete two questionnaires, the first addressing general characteristics such as age, sex and risk factors like smoking behavior, chronic medication and comorbidities, e.g. hypertension, diabetes mellitus, neoplasia, cardiovascular or pulmonary diseases. The second questionnaire specifically addressed the SARS-CoV-2 infection and asked for symptoms and their duration, possible hospitalization, and use of drugs and other remedies to relieve COVID-19 symptoms. A symptom diary covering 16 symptoms and a period of 25 days was also included. The first day of the observation period was defined as either the first day with COVID-19 symptoms, or the day of the positive test when patients had no symptoms at that time. The following symptoms were specified: headache, body aches, fatigue, dry cough, productive cough, sore throat, dyspnea, loss of taste, loss of smell, fever (with a blank field for entering the temperature), diarrhea, nausea, abdominal pain, vomiting, skin manifestation, conjunctivitis. The participants could check “yes” or “no” for each symptom on each day of the observation period.

Requirements for inclusion in the analysis were the return of the signed consent form and of both questionnaires with complete answers. We invited 3840 individuals to participate in the COVER study. Of these, 983 individuals (25.6%) consented to participate in the study. 74.4% of the invited individuals refused or did not answer. Finally, 710 individuals (18.5%) who provided complete questionnaires could be included. Overall we consider this to be a random sample.

The questionnaires were pseudonymized. To build the database, we employed double data entry into Excel tables by two different study team members for self-checking and to avoid errors in data transmission from questionnaires to database. Quality control was assured by merging both Excel tables and thereby detecting incongruent data in two corresponding cells in both tables.

Study participants were asked to note the medicines they used to treat SARS-CoV-2 infection and to provide the exact reasons for use. That included OTC drugs, as well as prescription drugs and medical devices. Moreover, patients were asked about other remedies they used to relieve their symptoms, e.g. teas, inhalation and self-made formulations like cough syrups or soups. We analyzed all individual active ingredients contained in the drugs and remedies mentioned and classified them into groups describing their main pharmacological effect or indication. We did not classify the drugs according to the ATC-code, because not every given answer could be clearly assigned. The participants were free to choose their own wording for reasons of use. We categorized each stated reason for use into thematic groups. The statistical analyses of the data were performed using IBM SPSS Statistics Version 28.0.1. For metric variables (age and BMI), mean values were calculated and differences between men and women were analyzed using the t-test for independent samples. Nominal values are presented as absolute numbers and relative frequencies [%], and group differences were compared using the Pearson chi-square test. In small samples with an expected frequency < 5, Fisher's exact test was used instead. Pearson correlation coefficients and respective statistical significances were determined for bivariate correlations. All statistical tests used where two-tailed.

## Results

### Characteristics of the study population

During recruitment period, 3840 individuals were invited to participate in the COVER study. The number represents 71.5% of the infection cases reported in the district of Vorpommern-Greifswald during this period. The remaining 28.5% were not invited because of exclusion criteria, or because contact information was missing. In total, 710 individuals completed and returned both questionnaires. Their data were transferred into a database for analysis.

Of the total of 710 study participants, 59.9% were women and 40.1% were men. Mean age was 50.9 years (range 18–98 years). 60.5% had a body mass index (BMI) ≥ 25 kg/m^2^ and 68.9% reported having at least one comorbidity. The most frequently reported comorbidities were hypertension (37.2%), allergies (20.3%) and joint diseases (18.7%). A BMI ≥ 25 kg/m^2^ as well as the presence of cardiovascular disease, diabetes mellitus and stroke were reported more frequently in men than women. Table 1 summarizes the most important characteristics of the study population. Part of these data have already been reported in the original publication of the course and symptoms of COVID-19 in this particular outpatient population^11^.

**Table 1:** Characteristics of the study population (taken in part from^11^).

Parameter	All (n = 710)	Women (n = 425)	Men (n = 285)	*p*-value
Age in years*	50.9 (18–98)	50.4 (18–98)	51.7 (18–85)	0.245
Age ≥ 60 years	229 (32.5%)	119 (28.2%)	110 (38.9%)	**0.003**
BMI in kg/m^2^**	27.5 (16.3–56.7)	27.2 (16.3–56.7)	28.1 (18.9–43.6)	**0.027**
Overweight (BMI ≥ 25 kg/m^2^)	430 (61.5%)	230 (54.9%)	200 (71.4%)	**< 0.001**
Current smoker	116 (16.3%)	71 (16.7%)	45 (15.8%)	0.757
Former smoker	211 (29.7%)	91 (21.4%)	120 (42.1%)	** < 0.001**
Never smoker	383 (53.9%)	263 (61.9%)	120 (42.1%)	** < 0.001**
At least one comorbidity	489 (68.9%)	288 (67.8%)	201 (70.5%)	0.457
Hypertension	264 (37.2%)	147 (34.6%)	117 (41.1%)	0.082
Allergy	144 (20.3%)	95 (22.4%)	49 (17.2%)	0.106
Joint diseases	133 (18.7%)	78 (18.4%)	55 (19.3%)	0.769
Cardiovascular diseases	80 (11.3%)	33 (7.8%)	47 (16.5%)	** < 0.001**
Pulmonary diseases	64 (9.0%)	31 (7.3%)	33 (11.6%)	0.061
Diabetes mellitus	61 (8.6%)	26 (6.1%)	35 (12.3%)	**0.004**
Neoplasia	56 (7.9%)	28 (6.6%)	28 (9.8%)	0.121
Neurological diseases	50 (7.0%)	29 (6.8%)	21 (7.4%)	0.881
Liver diseases	49 (6.9%)	33 (7.8%)	16 (5.6%)	0.294
Kidney diseases	33 (4.6%)	18 (4.2%)	15 (5.3%)	0.587
Stroke	18 (2.5%)	6 (1.4%)	12 (4.2%)	**0.027**
Peripheral artery disease	14 (2.0%)	6 (1.4%)	8 (2.8%)	0.270

Values are shown as numbers (%), age and BMI as mean (minimum and maximum value). *p*-values describe group comparisons between men and women (Pearson chi-square test for frequencies; t-test for independent samples for age and BMI). *BMI* body mass index.

*n = 705 (283 men; 422 women). **n = 699 (280 men; 419 women). Bold: *p* ≤ 0.05.

We asked the participants to complete a symptom diary covering 16 symptoms and a period of 25 days (see^11^ for details). Seven hundred participants delivered diaries, most commonly reported symptoms were fatigue (79.6%), cough (productive and dry cough were summarized; 72.3%) and headache (69.6%). Of the participants, 4.9% were asymptomatic and 5.2% (n = 695) required hospitalization. The largest proportion of participants reported a duration of symptoms of 25 days (30.2%; n = 700). Our population also showed sex-specific differences in the course of COVID-19 disease, with symptoms lasting an average of 17.5 days in women and 13.5 days in men (*p* < 0.001). Headaches, loss of smell and taste, sore throat or gastrointestinal complaints occurred more frequently in women^11^.

### Use of pharmaceutical products and other remedies

Out of 710 study participants, 523 (73.7%) reported usage of drugs and other remedies to treat symptoms due to SARS-CoV-2 infection. Women reported a higher rate than men (80.0% vs. 64.2%, *p* < 0.001). A total of 1282 finished dosage products and other remedies were reported. The number of products and remedies per participant ranged from one to eleven, with a mean of 2.6 in women and 2.2 in men (n = 523; *p* = 0.027). In total, we identified 213 different ingredients. Several of the reported products and remedies contained more than one ingredient, adding up to 1453 reported utilizations of these ingredients. Ingredients reported by at least ten participants are shown in Table 2. The largest group (n = 531 reported utilizations) were analgetic, antiphlogistic and antipyretic drugs (e.g. ibuprofen, acetaminophen, metamizole, acetylsalicylic acid). Second, 406 out of 1453 reported utilizations belonged to phytopharmaceuticals, herbal (e.g. home-grown or self-collected herbs, onion juice or lemon) and animal products (e.g. honey, milk, chicken broth), including products such as teas or self-made home remedies. Vitamins and minerals represented the third largest group (141 out of 1453). Considering the most frequently reported individual ingredients in each of these three classes, 25.5% of the 710 study participants used ibuprofen, 20.6% acetaminophen, 13.7% metamizole and 10.0% acetylsalicylic acid. Furthermore, 4.4% of participants mentioned preparations of eucalyptus, 3.8% thyme, 3.7% lemon and 3.3% primrose. Among vitamins and minerals 6.3% of the participants reported vitamin C, 3.1% vitamin D and 2.7% zinc. Furthermore, many study participants reported drugs against cough or rhinitis, for example with acetylcysteine (4.2%), xylometazoline (4.1%), or containing sodium chloride or sea-salt (3.1%).

**Table 2:** Most frequently reported ingredients.

Active ingredients	All (n = 710)	Women (n = 425)	Men (n = 285)	*p*-value
Ibuprofen	181 (25.5%)	122 (28.7%)	59 (20.7%)	**0.018**
Acetaminophen	146 (20.6%)	90 (21.2%)	56 (19.7%)	0.637
Metamizole	97 (13.7%)	69 (16.2%)	28 (9.8%)	**0.019**
Acetylsalicylic acid	71 (10.0%)	40 (9.4%)	31 (10.9%)	0.610
Ascorbic acid	45 (6.3%)	27 (6.4%)	18 (6.3%)	1.000
Eucalyptus oil	31 (4.4%)	20 (4.7%)	11 (3.9%)	0.709
Acetylcysteine	30 (4.2%)	19 (4.5%)	11 (3.9%)	0.710
Xylometazoline	29 (4.1%)	20 (4.7%)	9 (3.2%)	0.340
Thyme tea/oil/extract	27 (3.8%)	19 (4.5%)	8 (2.8%)	0.319
Lemon/lemon oil	26 (3.7%)	22 (5.2%)	4 (1.4%)	**0.013**
Pseudoephedrine	23 (3.2%)	15 (3.5%)	8 (2.8%)	0.670
Vitamin D	22 (3.1%)	13 (3.1%)	9 (3.2%)	1.000
Caffeine	21 (3.0%)	13 (3.1%)	8 (2.8%)	1.000
Primrose root/primrose flower extract/primrose with calyx powder	21 (3.0%)	18 (4.2%)	3 (1.1%)	**0.021**
Bromhexine	19 (2.7%)	12 (2.8%)	7 (2.5%)	0.817
Zinc/zinc oxide	19 (2.7%)	13 (3.1%)	6 (2.1%)	0.487
Ivy leaf extract	16 (2.3%)	8 (1.9%)	8 (2.8%)	0.447
Chlorphenamine	15 (2.1%)	8 (1.9%)	7 (2.5%)	0.791
Verbena powder/extract	15 (2.1%)	12 (2.8%)	3 (1.1%)	0.120
Gentian extract/gentian root extract/powder	15 (2.1%)	12 (2.8%)	3 (1.1%)	0.120
Elderflower powder/extract	15 (2.1%)	12 (2.8%)	3 (1.1%)	0.120
Menthol, levomenthol	15 (2.1%)	10 (2.4%)	5 (1.8%)	0.612
Sorrel herb powder	15 (2.1%)	12 (2.8%)	3 (1.1%)	0.120
NaCl, salt water	14 (2.0%)	12 (2.8%)	2 (0.7%)	0.055
Myrtle oil	14 (2.0%)	12 (2.8%)	2 (0.7%)	0.055
Sweet orange oil	14 (2.0%)	12 (2.8%)	2 (0.7%)	0.055
Ambroxol	13 (1.8%)	11 (2.6%)	2 (0.7%)	0.087
Dexpanthenol, panthenol	13 (1.8%)	10 (2.4%)	3 (1.1%)	0.261
Fruits, vegetables, vitamin-rich diet	13 (1.8%)	10 (2.4%)	3 (1.1%)	0.261
Cineole	12 (1.7%)	8 (1.9%)	4 (1.4%)	0.771*
Codeine	11 (1.5%)	7 (1.7%)	4 (1.4%)	1.000*
Ginger	11 (1.5%)	8 (1.9%)	3 (1.1%)	0.539*
Racemic camphor	11 (1.5%)	6 (1.4%)	5 (1.8%)	0.762*

Ingredients taken by ≥ 10 participants are shown as numbers (%). *p*-values describe group comparisons between men and women using the Pearson chi-square test.

*Fisher's exact test was used for group differences in samples with an expected frequency < 5. Bold: *p*-value ≤ 0.05.

### Reasons for the use of pharmaceutical products and other remedies

Because study participants reported reasons for using pharmaceutical products and remedies in their own wording, the answers were rather diverse and we summarized them in groups (Table 3). Forty percent of the n = 523 participants utilizing a pharmaceutical product or other remedies mentioned headache, the second most common reason were other types of pain such as myalgia or arthralgia (37.3%). Fever was reported by 23.9%, cough by 16.1% and rhinitis by 9.0%. Other reasons, e.g. medical advice, general feeling of illness or relief of symptoms, summed up to 9.2%. Thrombosis prophylaxis (3.4%), support of the night rest or sleep (3.1%), fatigue (2.3%), gastrointestinal symptoms like nausea (2.1%) and diarrhea (0.6%), or “strengthening” of the immune system (2.1%) were some of the less frequently reported reasons.

**Table 3:** Reasons for use of pharmaceutical products or other remedies against SARS-CoV-2-infection.

Headings for indicated reasons for use	All (n = 523)	Women (n = 340)	Men (n = 183)	*p*-value
Headache	209 (40.0%)	149 (43.8%)	60 (32.8%)	**0.015**
Myalgia/arthralgia/other kind of pain	195 (37.3%)	138 (40.6%)	57 (31.1%)	**0.037**
Fever	125 (23.9%)	79 (23.2%)	46 (25.1%)	0.668
Cough (dry or productive cough)	84 (16.1%)	61 (17.9%)	23 (12.6%)	0.134
Feeling of illness, relief of symptoms, support of recovery, prophylaxis, medical advice	48 (9.2%)	25 (7.4%)	23 (12.6%)	0.057
Rhinitis	47 (9.0%)	36 (10.6%)	11 (6.0%)	0.108
Other symptoms of upper respiratory tract infections	32 (6.1%)	23 (6.8%)	9 (4.9%)	0.450
Sore throat	20 (3.8%)	15 (4.4%)	5 (2.7%)	0.474
Thrombosis prophylaxis	18 (3.4%)	11 (3.2%)	7 (3.8%)	0.803
Aid to sleep	16 (3.1%)	6 (1.8%)	10 (5.5%)	**0.030**
Fatigue and fatigue-like symptoms	12 (2.3%)	10 (2.9%)	2 (1.1%)	0.231*
Symptoms of lower respiratory tract infections, dyspnea and impaired breathing	12 (2.3%)	10 (2.9%)	2 (1.1%)	0.231*
“strengthening” of the immune system	11 (2.1%)	7 (2.1%)	4 (2.2%)	1.000*
Nausea	11 (2.1%)	8 (2.4%)	3 (1.6%)	0.755*

Summarizing terms for reasons to apply pharmaceutical products and other remedies which study participants reported at least once. Values are shown as numbers (%). *p*-values describe group comparisons between men and women using the Pearson chi-square test.

*Fisher's exact test was used for group differences in samples with an expected frequency < 5. Bold: *p*-value ≤ 0.05.

### Correlations between pharmaceutical products and remedies and characteristics of the study population

We also analyzed correlations between the utilized number of pharmaceutical products and other remedies to treat the symptoms of SARS-CoV-2 infection and certain characteristics of the study population (age, BMI, number of comorbidities, long-term medication, duration and number of symptoms). A positive linear correlation was only found for the number of days with symptoms (r = 0.425; *p* < 0.001) and the number of different symptoms (r = 0.470; *p* < 0.001). Furthermore, participants with certain risk factors for a more severe course of disease (e.g. BMI ≥ 25 kg/m^2^, cardiovascular diseases, hypertension, diabetes mellitus or lung diseases, n = 515) were compared with those without the mentioned risk factors (n = 195). No significant differences were found in the usage of particular active substances or the number of pharmaceutical products and other remedies used between those two groups.

## Discussion

In this analysis, we determined the use of drugs and other remedies against symptoms of SARS-CoV-2 infection in outpatients. We assumed that the majority of participants were self-medicating with over-the-counter (OTC) drugs, however, we captured all drugs mentioned, both OTC as well as prescribed drugs. Our results show a hugh variety of utilized drugs and remedies and reasons for their use.

Overall, 340 women and 183 men out of 710 outpatients reported using drugs and other remedies to relieve COVID-19-symptoms. We identified 213 different ingredients, and utilization of ibuprofen (26%), acetaminophen (21%), metamizole (14%), and acetylsalicylic acid (10%) was most commonly reported. Phytopharmaceuticals, herbal and animal products as well as vitamins and minerals were also frequently reported.

Except for a minimum age of 18 years and a current positive PCR test, there were no other inclusion or exclusion criteria for the recruitment of study participants. More women than men consented into participation. The proportion of COVID-19 patients with comorbidities (68.9%) in our cohort is slightly higher than in published data for individuals with chronic diseases in Germany, e.g. 49.2% in surveys by the Robert Koch Institute^12^. Most comorbidities (e.g. cardiovascular diseases) were more commonly reported by men, similar sex-specific differences are reported in the published data^12,13^.

COVID-19 symptoms were more frequent and longer lasting in women. 30.2% of participants reported a symptom duration of 25 days. However, because the questionnaire only included a 25-day symptom diary, longer symptom duration could not be detected with our questionnaire. The prevalence of individual symptoms varies considerably in the published data, possibly because of differences in study designs, symptom descriptions, study populations, utilized therapies, and the respective infection processes at different locations and times of COVID-19 study conduct.

Our results suggest that more women than men (80.0% vs. 64.2%, *p* < 0.001) tried to alleviate COVID-19-symptoms by using pharmaceutical products and other remedies. Published data for self-medication use in Germany, regardless of a specific disease, show a comparable proportion between men and women^14^. Women also used a greater number of products or remedies against their symptoms. Except the longer average duration of symptoms in women, no reasons for these differences can be deduced from our study results. Published data suggests that contributing factors may be a greater awareness of health and body, a broader knowledge of health issues and a stronger connection to the healthcare system^15–17^. Overall, the participants stated a variety of pharmaceutical products and remedies and reasons for use. Especially analgesic and antipyretic agents, followed by phytopharmaceuticals as well as vitamins and minerals, have been used very frequently. In particular, these products and remedies were utilized against general symptoms of respiratory diseases like headache, myalgia, arthralgia, fever and cough which were also frequently mentioned in the symptom diary. Specific symptoms like loss of smell or taste and dyspnea were commonly reported, but rarely were reported as a reason for drug use, probably due to low availability of suitable drugs. The frequency distribution of pharmaceutical products and remedies corresponds approximately to the indication groups of the non-prescription drugs with the highest sales in Germany which include the respiratory system (e.g. remedies against cold), the alimentary system and metabolism (e.g. vitamins and minerals) and the nervous system (e.g. analgesic drugs)^18^. Published data for self-medication use in Germany also show a frequent use of products from these Anatomical-Therapeutic-Chemical-groups^19^.

With regard to frequently reported symptoms such as headache, myalgia, fever or cough, a large frequency of utilization of analgesic, antipyretic and antitussive or expectorant drugs was expected. The most frequently mentioned active ingredient was ibuprofen (25.5%; n = 710), which is the second most prescribed drug in Germany despite being available in OTC-formulations^20^. Originally, the questionnaires for drug use were also developed to prove the public assumption at that early stage of the pandemia that ibuprofen and ACE-inhibitors carried a risk for more severe courses of the disease. As described in the original publication, we could exclude these drugs as risk factors for higher infection rates or as cause for a more severe course^11^. Several further studies were published which could not prove an assumed increased risk, for example due to ibuprofen^21^. Metamizole (13.7%) was also frequently used. Metamizole is only available on prescription in Germany and has a restricted therapeutic indication, however it was the most frequently prescribed drug in 2020 in Germany^20^. Thus, the large number of patients who took metamizole in our cohort is on first sight not surprising, but in terms of drug therapy safety, the finding is alarming, because many of these patients were not under regular medical control. The second most frequently prescribed group of active ingredients in our study were phytopharmaceuticals, herbal and animal products. They include many different drugs used against colds and flu, cough, sore throat or sinusitis. Eucalyptus, thyme and primrose were common ingredients. Combination medicines containing several active ingredients were also frequently used here.

In addition, we analyzed correlations between number of reported pharmaceutical products and other remedies and characteristics of our cohort such as age, BMI, comorbidities and course of disease. Only the duration of symptomatic course and the number of different symptoms were moderately correlated with the frequency of usage.

Other studies which determined drug use and self-medication to relieve COVID-19 symptoms are not easily comparable to ours. Several studies conducted in countries other than Germany focused on self-medication during the COVID-19 pandemic, but often without confirmation of the infection. Some studies analyzed self-medication in the context of COVID-19 prevention^7–10^. The frequent use of antibiotics, antimalarial drugs (e.g. hydroxychloroquine), and ivermectin for self-medication in these studies is conspicuous. During the initial time of the SARS-CoV-2 pandemic, the knowledge about the course of the disease and treatment possibilities was very limited and characterized by uncertainty and missinformation. In our study, antimalarial and anthelminthic drugs were not, and antibiotics were only rarely mentioned. Comparable to published data, we found a frequent use of vitamins and minerals, phytopharmaceuticals and analgesic and antipyretic drugs. However, comparisons with other countries, especially non-European Union countries, are difficult, due to different legal requirements, awareness and accessibility of medicines.

## Limitations

However, we were limited in analyzing the dosage and course of drug use because of the questionnaire design. The participants were asked to indicate the number of days they utilized each drug, but we could not infer the exact day during the infection, so we were not able to assign a chronological relationship between symptoms course or comedication. Based on the observational cohort study design, no causal relationships could be determined between the course of disease and self-medication. Moreover, neither the efficacy of utilized drugs and remedies, nor participants’ satisfaction regarding symptom relieve or duration of infection could be determined. A huge number of different pharmaceutical products and remedies were used, so the frequency of many products and remedies was too small to determine causality or significance with regard to the impact of individual active ingredients. The questionnaires were initially created to collect data on the course of disease and risk factors for hospitalization in outpatients for the COVER study^11^. Due to the extensive number of questions in the questionnaires, to further analyze aspects such as self-medication seemed appropriate. However, this also resulted in some limitations for the evaluation as described. Other aspects are for example possible selection bias with regard to comorbidities, risk factors or symptomatic course of disease. Also, there were no restrictions regarding the description of drugs and other remedies or the reasons for use, it is difficult to summarize them. As this is an observational study based on questionnaires, there was no randomization or intervention. Therefore, it is not possible to establish direct causal relationships between individual parameters.

## Conclusion

The main focus of our analysis was to determine and describe drugs and remedies taken to alleviate the symptoms of a confirmed SARS-CoV-2 infection in German outpatients. Almost three quarters of participants reported the utilization of a wide variety of drugs and remedies. We were able to show that the majority used active ingredients which are also typically used to alleviate colds, flu or other respiratory infections. Further studies to examine a possible causal relationship between OTC drugs and course of COVID-19 disease would be useful for medical and pharmaceutical advice.

## Data Availability

The COVER database contains sensitive information about the health of the study participants that cannot be deposited openly. However, individual-level data that support the findings of this study could be obtained from corresponding author following an approval of the data steering committee of the University Medicine Greifswald.
